# Comparison of survival between right‐sided and left‐sided colon cancer in different situations

**DOI:** 10.1002/cam4.1401

**Published:** 2018-03-13

**Authors:** Miao‐Zhen Qiu, Wen‐Tao Pan, Jun‐Zhong Lin, Zi‐Xian Wang, Zhi‐Zhong Pan, Feng‐Hua Wang, Da‐Jun Yang, Rui‐Hua Xu

**Affiliations:** ^1^ Department of Medical Oncology Sun Yat‐Sen University Cancer Center Guangzhou China; ^2^ State Key Laboratory of Oncology in South China Guangzhou China; ^3^ Collaborative Innovation Center for Cancer Medicine Guangzhou China; ^4^ Department of Experimental Research Sun Yat‐Sen University Cancer Center Guangzhou China; ^5^ Department of Colorectal Surgery Sun Yat‐Sen University Cancer Center Guangzhou China

**Keywords:** Left‐sided colon cancer, right‐sided colon cancer, surveillance epidemiology and end results, stage, survival

## Abstract

Mountain of studies has showed that right‐sided colon cancer (RSCC) and left‐sided colon cancer (LSCC) have different clinical presentation and biologic features and should be considered as two distinct disease entities. The survival difference between RSCC and LSCC remains controversial. Using Surveillance, Epidemiology, and End Results (SEER) database, we identified colon adenocarcinoma patients from 2004 to 2013. The 5‐year cause‐specific survival (CSS) was our primary endpoint. All statistical analyses were performed using the Intercooled Stata 13.0. All statistical tests were two‐sided. The study included 95,847 (58.72%) RSCC and 67,385 (41.28%) LSCC patients. RSCC patients were older, more often females, more Caucasian, more unmarried, more advanced T and N stage, larger tumor sizes, and more poorly differentiated tumor, while LSCC patients had more stage IV diseases. Location was an independent prognostic factor in the multivariable analysis. Compared with RSCC patients, the hazard ratio for LSCC was 0.87, 95% CI: 0.85–0.89 *P *<* *0.001. There was no survival difference between RSCC and LSCC in the following situations: older than 68 years old, T3–4, N0, poorly differentiated, and undifferentiated diseases. We firstly reported that RSCC patients had a better prognosis than LSCC in mucinous adenocarcinoma/signet ring cell carcinoma patients. RSCC patients also had a better prognosis than LSCC in stage II disease. There is a need for further subdivisions when analyzing the survival difference between RSCC and LSCC patients. RSCC had lower mortality rate than LSCC in stage II disease and mucinous adenocarcinoma/signet ring cell carcinoma patients.

## Introduction

Colon cancer is among the leading causes of cancer‐related deaths all over the world 1, 2. Mountain of studies has showed that right‐sided colon cancer (RSCC) and left‐sided colon cancer (LSCC) should be considered as two distinct disease entities 3, 4, 5, 6. Differences in embryologic development, clinical presentation, patient demographics, and tumor biology between RSCC and LSCC have been clearly reported in the literatures 3, 5, 6, 7, 8, 9. It is well known that RSCC arises from the embryonic midgut and is perfused by the superior mesenteric artery, while LSCC originates from the hindgut and is served by the inferior mesenteric artery 6. Moreover, the capillary network surrounding the LSCC is multilayered, whereas that of the LSCC is single‐layered, possibly relating to the greater water absorption and electrolyte transport capacity of the former 10. The difference in anatomic structure may partly explain the different clinical presentation between RSCC and LSCC, such as more advanced T stage with severe symptoms (passage trouble or abdominal mass) in RSCC patients 7, 11.

Whether the biologic and clinical differences between RSCC and LSCC have translated into clinically meaningful prognostic difference is still controversial. Although accumulating evidences suggest that RSCC patients have a worse prognosis than LSCC patients 4, 5, 12, 13, 14, 15, 16, Weiss JM et al. 15 used the Medicare beneficiaries of colon adenocarcinoma to compare survival between RSCC and LSCC patients by stage and found that there was no overall difference in 5‐year mortality between RSCC and LSCC patients. Their further analysis showed that stage II RSCC had lower mortality, while stage III RSCC had higher mortality than LSCC 15. Except for stage, are there other factors affecting the survival comparison between RSCC and LSCC patients? In this study, we used data from Surveillance, Epidemiology, and End Results (SEER) cancer registry program of individuals diagnosed with colon adenocarcinoma from 2004 to 2013 to compare the survival and clinicopathologic features between RSCC and LSCC patients in different situations.

## Methods

### Statistics

The patients’ demographic and tumor characteristics were summarized with descriptive statistics. Comparisons of categorical variables between right and left colon cancer patients were performed using the chi‐squared test, and continuous variables were compared using Student's *t* test. The primary endpoint of this study was 5‐year cause‐specific survival (CSS), which was calculated from the date of diagnosis to the date of cancer‐specific death. Deaths attributed to colon cancer were treated as events, and deaths from other causes were treated as censored observations. Survival function estimation and comparison between RSCC and LSCC were performed using Kaplan–Meier estimates and the log‐rank test. The independence of the prognostic effect of location was evaluated by adjusting for other known factors including age at diagnosis and tumor stage. The multivariate Cox proportional hazard model was used to evaluate the hazard ratio (HR) and the 95% confidence interval (CI) for all the prognostic factors. All of statistical analyses were performed using the Intercooled Stata 13.0 (Stata Corporation, College Station, TX). Statistical significance was set at two‐sided *P *<* *0.05.

### Database

The SEER database is the largest publicly available cancer dataset. It is a population‐based cancer registry across several disparate geographic regions. The SEER research data include cancer incidence and prevalence as well as demographic information tabulated by age, sex, race/ethnicity, year of diagnosis, and geographic region. The dataset we used for this analysis was Surveillance, Epidemiology, and End Results (SEER) Program (http://www.seer.cancer.gov) Research Data (1973–2013).

### Outcome variables

The anatomic subsites of the left colon and right colon were categorized according to the International Classification of Diseases for Oncology, third edition (ICD‐0‐3) topography codes. RSCC was identified with the following SEER cancer site codes: cecum (ICD‐0‐3 code C18.0), ascending colon (Code C18.2), hepatic flexure (Code C18.3), and transverse colon (Code C18.4). LSCC was identified with codes: splenic flexure (Code C18.5), descending colon (code C18.6), and sigmoid colon (code C18.7). Rectosigmoid (code C19.9) was excluded from the analysis.

For the Race/Ethnicity, we reclassified the patients into four groups: “Caucasian” (Race/Ethnicity code, 1), “African American” (Race/Ethnicity code, 2), “Asian” (Race/Ethnicity code, 4–6, 8–17 and 96), and “Others” (The rest code).

In this article, only adenocarcinoma patients were enrolled (SEER histology codes: signet ring cell, 8490; mucinous adenocarcinoma, 8480 and 8481; other adenocarcinoma: 8140–8147, 8210–8211, 8220–8221, 8260–8263, and 8570–8576).

### Patient population

The study population was based on the SEER cancer registry. Since the American Joint Committee on Cancer (AJCC) 7th Tumor‐Node‐Metastasis (TNM) staging system was released in 2010 and if we used this staging system, there would be no 5‐year survival due to insufficient follow‐up, so we picked up the AJCC 6th TNM staging systems. Meanwhile, since the AJCC 6th TNM staging system was released in 2004, we selected patients from 2004 to 2013.

All patients had active follow‐up and the survival month was over 1 month. Patients were excluded if they had more than one primary cancer, but colon cancer was not the first one or had unknown cause of death. AJCC 6th TNM staging systems were used for the staging. We excluded patients whose TNM stage was unknown.

## Results

### Patient baseline characteristics

The study identified 163,232 colon adenocarcinoma patients including 80,599 (49.38%) men and 82.633 (50.62%) women. Of these patients, 95,847 (58.72%) were RSCC and 67,385 (41.28%) were LSCC. The mean age of the whole population was 67.28 ± 13.61 (Mean ± SD) with a median age of 68 years old.

### Clinicopathologic features of patients with RSCC and LSCC

Table 1 showed the basic features between these two groups of patients. The proportion of men was significantly higher in LSCC than in RSCC patients, *P *<* *0.001. The median age of LSCC patients was significantly younger than RSCC patients, 64 and 71 years old, respectively, *P *<* *0.001. More LSCC patients were married. For the TNM stage, LSCC patients had higher percentage of stage I and IV diseases. Poorly differentiated and undifferentiated adenocarcinoma or mucinous adenocarcinoma/signet ring cell carcinoma was less common in LSCC than in RSCC patients. LSCC was more likely to be detected at a smaller tumor size than RSCC patients (median tumor size: 40 mm vs. 45 mm), *P *<* *0.001. RSCC patients received more surgery and less radiation than LSCC patients. For those receiving operation, RSCC had more lymph nodes resected and fewer positive lymph nodes.

**Table 1:** Comparison of clinicopathological features between right‐sided and left‐sided colon cancer

	Right‐sided colon (%)	Left‐sided colon (%)	*P* values
Gender			
Male	44,112 (46.02)	36,487 (54.15)	
Female	51,735 (53.98)	30,898 (45.85)	<0.001
Age (Mean ± SD)	69.49 ± 13.27	64.15 ± 13.48	<0.001
Ethnicity			
Caucasian (%)	76,668 (79.99)	51,372 (76.24)	
African American	12,475 (13.02)	8169 (12.12)	
Asian	5449 (5.69)	6457 (9.58)	
Others	1255 (1.31)	1387 (2.06)	<0.001
Married status			
Married	50,435 (52.62)	37,786 (56.07)	
Unmarried	41,308 (43.10)	26,265 (38.98)	
Unknown	4104 (4.28)	3334 (4.95)	<0.001
AJCC 6th TNM stage			
I	21,561 (22.5)	18,638 (27.66)	
II	30,186 (31.49)	17,058 (25.31)	
III	27,015 (28.19)	18,602 (27.61)	
IV	17,085 (17.83)	13,087 (19.42)	<0.001
AJCC 6th T stage			
T0	13 (0.01)	10 (0.01)	
T1	12,315 (12.85)	14,816 (21.99)	
T2	13,675 (14.27)	7859 (11.66)	
T3	51,953 (54.20)	32,630 (48.42)	
T4	15,033 (15.68)	9736 (14.45)	
TX	2858 (2.98)	2334 (3.46)	<0.001
AJCC 6th N stage			
N0	55,463 (57.87)	39,355 (58.4)	
N1	22,642 (23.62)	16,687 (24.76)	
N2	15,984 (16.68)	9868 (14.64)	
NX	1758 (1.83)	1475 (2.19)	<0.001
Histology			
Other adenocarcinoma	82,848 (86.44)	62,884 (93.32)	
Mucinous adenocarcinoma	11,625 (12.13)	4055 (6.02)	
Signet ring cell	1374 (1.43)	446 (0.66)	<0.001
Grade			
Well differentiated	7688 (8.02)	6344 (9.41)	
Moderately differentiated	61,096 (63.74)	46,809 (69.47)	
Poorly differentiated	19,639 (20.49)	8385 (12.44)	
Undifferentiated	2257 (2.35)	829 (1.23)	
Unknown	5167 (5.39)	5018 (7.45)	<0.001
Lymph node resected (Mean ± SD)	17.97 ± 13.48	14.51 ± 13.69	<0.001
Positive lymph node (Mean ± SD)	10.01 ± 26.94	15.94 ± 34.22	<0.001
Tumor size (Mean ± SD, mm)	49.79 ± 36.82	43.80 ± 30.33	<0.001
Surgery			
Yes	90,084 (93.99)	62,675 (93.01)	
No	5708 (5.96)	4662 (6.92)	
Unknown	55 (0.06)	48 (0.07)	<0.001
Radiation			
Yes	1261 (1.32)	2037 (3.02)	
No	93,920 (97.99)	64,788 (96.15)	
Unknown	666 (0.69)	560 (0.83)	<0.001

AJCC, American Joint Committee on Cancer; SD, standard deviation; TNM, Tumor‐Node‐Metastasis.

### Survival analysis

The 5‐year CSS for the whole population was 69.3% (95% CI: 69.0–69.5%). There were 25,489 deaths (26.59%) in RSCC patients and 16,457 (24.42%) in LSCC patients. The 5‐year CSS was significantly longer in LSCC patients than in RSCC patients, 70.9% versus 68.1%, *P *<* *0.001.

The median age of the whole population was 68 years old. We therefore divided the patients into two groups according to the age: <69 years old (younger patients) and >68 years old (older patients). The younger patients had a significantly better 5‐year CSS than the older patients (71.2% vs. 67.2%, *P *<* *0.001; Table 2).

**Table 2:** Survival analysis in the whole population

	5‐year CSS	95% CI	*P* value
Gender			
Male	69.0%	68.6–69.4%	
Female	69.5%	69.1–69.9%	0.5552
Age			
<69	71.2%	70.8–71.6%	
>68	67.2%	66.8–67.6%	<0.001
Ethnicity			
Caucasian	70.1%	69.8–70.4%	
African American	62.0%	61.2–62.8%	
Asian	71.8%	70.8–72.7%	
Others	74.2%	71.7–76.5%	<0.001
Married status			
Married	72.1%	71.7–72.4%	
Unmarried	65.3%	64.8–65.7%	
Unknown	73.9%	72.7–75.1%	<0.001
Location			
Left‐sided colon	70.9%	70.5–71.3%	
Right‐sided colon	68.1%	67.7–68.4%	<0.001
AJCC 6th TNM stage			
I	93.6%	93.3–93.9%	
II	84.8%	84.4–85.2%	
III	68.3%	67.8–68.8%	
IV	13.1%	12.6–13.6%	<0.001
Histology			
Other adenocarcinoma	70.1%	69.8–70.4%	
Mucinous adenocarcinoma	65.3%	64.4–66.1%	
Signet ring cell	37.1%	34.5–39.7%	<0.001
Grade			
Well differentiated	83.0%	82.2–83.7%	
Moderately differentiated	72.8%	72.4–73.1%	
Poorly differentiated	55.9%	55.2–56.5%	
Undifferentiated	54.5%	52.3–56.8%	
Unknown	54.2%	53.1–55.3%	<0.001
Lymph node resected			
<12	61.5%	61.1–62.0%	
≥12	73.2%	72.8–73.5%	<0.001
Size			
≤43 mm	77.1%	76.7–77.4%	
>43 mm	63.4%	63.0–63.7%	<0.001
Surgery			
Yes	73.0%	72.7–73.2%	
No	9.3%	8.6–10.1%	
Unknown	26.8%	19.8–34.3%	<0.001
Radiation			
Yes	46.0%	44.0–48.0%	
No	69.8%	69.5–70.1%	
Unknown	62.5%	59.1–65.8%	<0.001

AJCC, American Joint Committee on Cancer; CI, Confidence interval; CSS, Cause‐specific survival; TNM, Tumor‐Node‐Metastasis.

No doubt, the TNM stage was significantly correlated with survival. The 5‐year CSS was 93.6%, 84.8%, 68.3%, and 13.1% for patients from stage I to stage IV, respectively, *P *<* *0.001. For the histology subtypes, signet ring cells had worse 5‐year CSS than the mucinous adenocarcinoma and other adenocarcinoma. When we analyzed the 5‐year CSS in patients with different grades, we found that the survival became poorer as the tumor grades progressed from well to undifferentiated, 83.0% for well differentiated, 72.8% for moderately differentiated, 55.9% for poorly differentiated, and 54.2% for undifferentiated tumors, *P *<* *0.001.

### Multivariate analysis

Variables showing a trend for association with survival (*P *<* *0.05) were selected in the Cox proportional hazards model. Age, married status, ethnicity, location, TNM stage, histologic subtypes, grade, tumor size, as well as surgery were all independent prognostic factors in the multivariable analysis. Compared with RSCC patients, the HR for LSCC patients was 0.87, 95% CI: 0.85–0.89, *P *<* *0.001 (Table 3).

**Table 3:** Multivariate analysis

	Hazard ratio	95% CI	*P* value
Age			
<69	Reference		
>68	1.71	1.68–1.75	<0.001
Ethnicity			
Caucasian	Reference		
African American	1.17	1.14–1.20	<0.001
Asian	0.87	0.84–0.91	<0.001
Others	1.06	0.98–1.15	0.172
Married status			
Married	Reference		
Unmarried	1.23	1.21–1.26	<0.001
Unknown	1.04	0.98–1.09	0.167
Location			
Right‐sided colon	Reference		
Left‐sided colon	0.87	0.85–0.89	<0.001
AJCC 6th TNM stage			
I	Reference		
II	2.13	2.03–2.24	<0.001
III	5.03	4.81–5.27	<0.001
IV	23.03	22.01–24.11	<0.001
Histology			
Other adenocarcinoma	Reference		
Mucinous adenocarcinoma	1.09	1.05–1.12	<0.001
Signet ring cell	1.41	1.32–1.51	<0.001
Grade			
Well differentiated	Reference		
Moderately differentiated	1.15	1.10–1.21	<0.001
Poorly differentiated	1.61	1.54–1.69	<0.001
Undifferentiated	1.76	1.64–1.90	<0.001
Unknown	1.27	1.20–1.34	<0.001
Size			
≤43 mm	Reference		
>43 mm	1.18	1.15–1.20	0.005
Surgery			
No	Reference		
Yes	0.56	0.42–0.77	<0.001
Unknown	0.85	0.65–1.12	0.252
Radiation			
Yes	Reference		
No	0.98	0.48–2.00	0.95
Unknown	1.22	0.60–2.47	0.587

AJCC, American Joint Committee on Cancer; CI, Confidence interval; TNM, Tumor‐Node‐Metastasis.

### Survival difference between RSCC and LSCC in different situations

We further compared the survival difference between RSCC and LSCC in different situations (Table 4). We found that LSCC patients had better prognosis than RSCC in both men and women, younger patients, all ethnicity subgroups, different married status, well and moderately differentiated adenocarcinoma patients and also in all the tumor sizes, and in patients receiving different therapies.

**Table 4:** Survival difference between RSCC and LSCC in different situations

	Right‐sided colon	Left‐sided colon	*P* value
Gender			
Male	67.8% (67.3–68.3%)	70.7% (70.2–71.3%)	<0.001
Female	68.5% (68.1–67.0%)	71.4% (70.8–72.0%)	<0.001
Age			
<69	69.1% (68.6–69.6%)	73.5% (73.0–74.0%)	<0.001
>68	67.5% (67.1–68.0%)	67.1% (66.4–67.7%)	0.5084
Ethnicity			
Caucasian	69.1% (68.9–69.7%)	71.9% (71.4–72.3%)	<0.001
African American	61.6% (60.5–62.6%)	63.0% (61.8–64.3%)	0.0041
Asian	70.8% (69.4–72.2%)	74.2% (72.9–75.5%)	<0.001
Others	68.1% (64.8–71.2%)	73.1% (70.2–75.9%)	0.0134
Married status			
Married	70.6% (70.2–71.1%)	74.0% (73.5–74.5%)	<0.001
Unmarried	64.9% (64.3–65.4%)	65.9% (65.2–66.6%)	<0.001
Unknown	71.2% (69.4–72.8%)	77.3% (75.5–79.0%)	<0.001
AJCC 6th TNM stage			
I	92.8% (92.4–93.2%)	94.6% (94.2–95.0%)	<0.001
II	85.5% (85.0–86.0%)	83.7% (83.0–84.3%)	<0.001
III	64.9% (64.2–65.6%)	73.4% (72.6–74.2%)	<0.001
IV	11.2% (10.6–11.9%)	16.2% (15.4–17.0%)	<0.001
AJCC 6th T stage			
T1	84.3% (83.6–85.0%)	89.9% (89.4–90.5%)	<0.001
T2	90.7% (90.1–91.2%)	91.0% (90.2–91.7%)	0.4867
T3	70.5% (70.0–71.0%)	70.5% (69.9–71.0%)	0.053
T4	37.1% (36.1–38.1%)	40.6% (39.3–41.8%)	<0.001
AJCC 6th N stage			
N0	83.8% (83.5–84.2%)	83.2% (82.7–83.6%)	0.1428
N1	59.5% (58.7–60.2%)	64.4 (63.5–65.2%)	<0.001
N2	33.8% (32.9–34.6%)	44.0% (42.8–45.2%)	<0.001
Histology			
Other adenocarcinoma	68.8% (68.4–69.2%)	72.1% (71.6–72.5%)	<0.001
Mucinous adenocarcinoma	67.3% (66.3–68.3%)	60.0% (58.2–61.7%)	<0.001
Signet ring cell	39.4% (36.3–42.4%)	31.0% (26.0–36.1%)	0.0034
Grade			
Well differentiated	82.1% (81.0–83.0%)	84.2% (83.2–85.3%)	0.0011
Moderately differentiated	72.5% (72.1–72.9%)	73.4% (72.9–73.8%)	<0.001
Poorly differentiated	56.2% (55.4–57.0%)	55.7% (54.5–57.0%)	0.0813
Undifferentiated	54.9% (52.2–57.5%)	54.0% (49.6–58.2%)	0.5098
Unknown	47.7% (46.2–49.3%)	61.2% (59.6–62.7%)	<0.001
Lymph nodes resected			
<12	55.6% (54.9–56.3%)	67.1% (66.4–67.7%)	<0.001
≥12	72.9% (72.5–73.3%)	73.9% (73.4–74.5%)	<0.001
Tumor size			
≤43 mm	76.6% (76.1–77.0%)	77.7% (77.1–78.2%)	<0.001
>43 mm	62.0% (61.6–62.5%)	65.4% (64.8–66.0%)	<0.001
Surgery			
Yes	71.7% (71.4–72.1%)	75.0% (74.6–75.4%)	<0.001
No	7.3% (6.4–8.2%)	12.8% (11.5–14.1%)	<0.001
Unknown	31.3% (16.5–47.3%)	35.7% (20.6–51.1%)	0.9440
Radiation			
Yes	33.6% (30.5–36.6%)	53.8% (51.3–56.4%)	<0.001
No	68.7% (68.4–69.1%)	71.7% (71.2–72.1%)	<0.001
Unknown	59.9% (55.2–64.3%)	65.9% (60.9–70.4%)	0.0287

AJCC, American Joint Committee on Cancer; LSCC, Left‐sided colon cancer; RSCC, Right‐sided colon cancer; TNM, Tumor‐Node‐Metastasis.

There was no significant difference between RSCC and LSCC in older patients or in those with poorly differentiated or undifferentiated adenocarcinoma. Meanwhile, no survival difference between RSCC and LSCC in T2, T3 or N0 disease was found.

**Figure 1:**
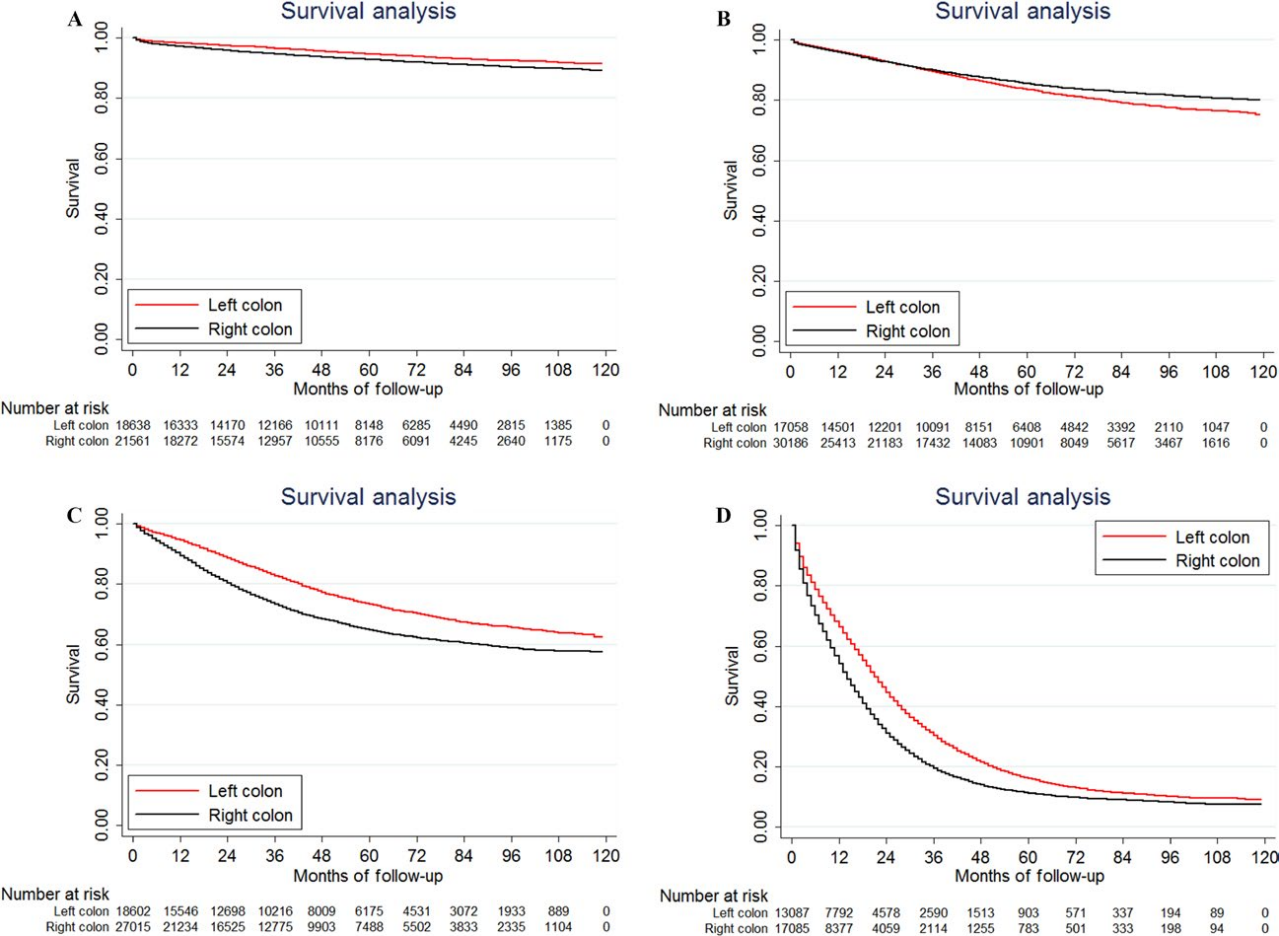
Kaplan–Meier survival estimates for patients with right‐sided and left‐sided colon cancer in (A) stage I disease; (B) stage II disease; (C) stage III disease; and (D) stage IV disease.

**Figure 2:**
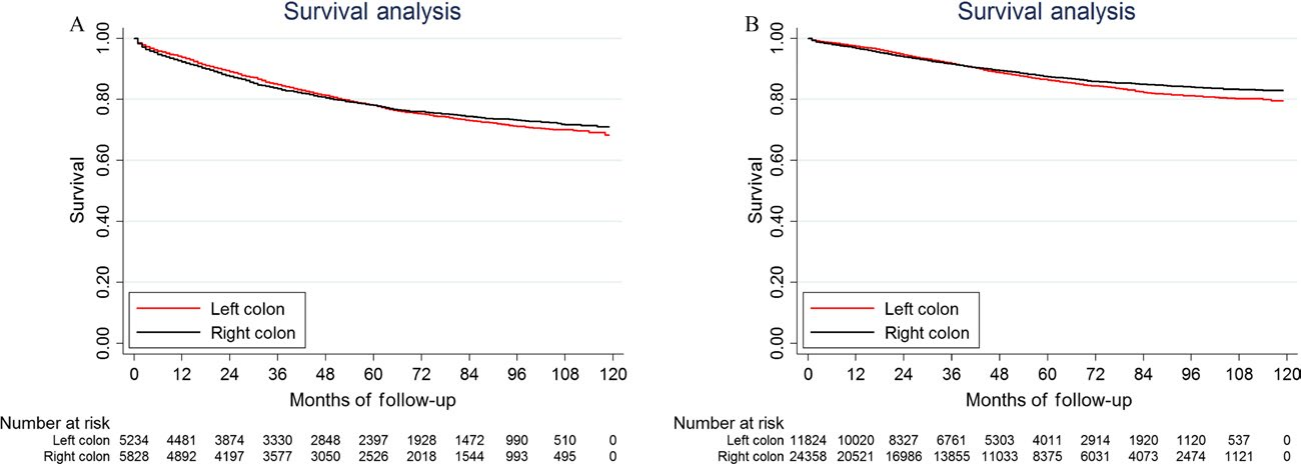
Kaplan–Meier survival estimates for stage II patients with right‐sided and left‐sided colon cancer when the number of resected lymph nodes was <12 (A); over 11 (B).

For patients with different TNM stages, LSCC had a better prognosis than RSCC except for stage II disease (Fig. 1). Surprisingly, the survival trend reversed in stage II disease, 83.7% and 85.5% for LSCC and RSCC patients, respectively, *P *<* *0.001. As we know that when the resected number of lymph nodes was <12, it may lead to inappropriate staging, especially for stage II disease. To better understand the survival difference in stage II disease, we analyzed the survival difference between RSCC and LSCC when resected lymph nodes were less 12 and over 11, respectively (Fig. 2). We found that there was no significant survival difference between these two groups when the resected number of lymph nodes was less 12, *P *=* *0.7829 (Fig. 2A). When the resected number of lymph nodes was over 11, stage II RSCC patients had significantly better prognosis than LSCC patients, *P *=* *0.0228 (Fig. 2B).

**Figure 3:**
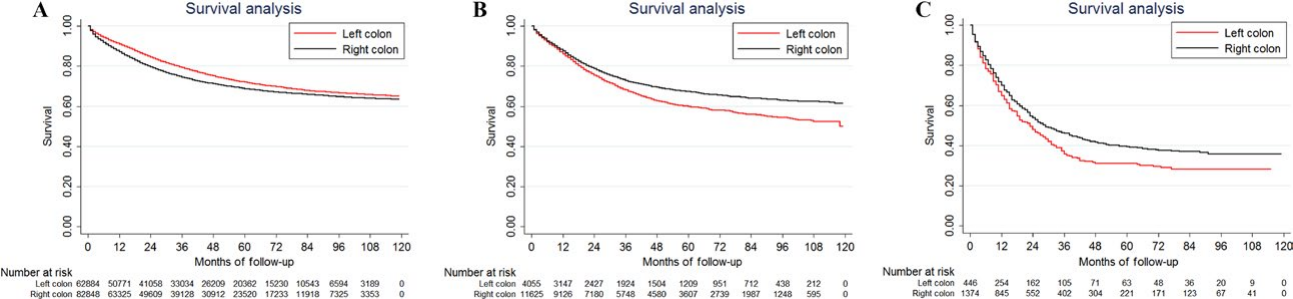
Kaplan–Meier survival estimates for patients with right‐sided and left‐sided colon cancer in (A) Other adenocarcinoma; (B) Mucinous adenocarcinoma; and (C) Signet ring cell carcinoma.

Moreover, LSCC patients also had a poorer survival than RSCC when the histology subtypes were mucinous adenocarcinoma or signet ring cell carcinoma (Fig. 3).

### Clinicopathologic features of stage II patients between RSCC and LSCC patients

To understand why LSCC patients had poorer survival than RSCC in stage II diseases, we compared the clinicopathologic features of stage II patients between RSCC and LSCC (Appendix S1). Overall, the clinicopathologic features between RSCC and LSCC were similar in stage II disease and in the whole population. Except that LSCC patients had more T4 diseases than RSCC in stage II while in the whole population, LSCC had less T4 diseases.

### Clinicopathologic features of mucinous adenocarcinoma/signet ring cell carcinoma patients between RSCC and LSCC patients

Similarly, we compared the clinicopathologic features of mucinous adenocarcinoma/signet ring cell carcinoma patients between RSCC and LSCC patients (Appendix S2). Compared with the whole population, more RSCC patients had stage I disease in mucinous adenocarcinoma/signet ring cell carcinoma patients.

## Discussion

A number of studies have been carried out in different regions of the world to describe the differences between RSCC and LSCC. Regarding the difference in biologic behavior and clinical presentation, RSCC and LSCC were suggested to be considered as two disease entities 5, 9, 15, 17. In this study, we found that the relationship between clinicopathologic features and tumor location in colon cancer was not straightforward. Specifically, RSCC patients not only had some adverse features, such as older, more unmarried, more advanced T and N stage, larger tumor sizes, and more poorly differentiated tumor which were similar to the previous reports 6, 11, 13, 15, 18, 19, but also had some good features, including less metastasis diseases and fewer numbers of positive lymph nodes. Most previous comparisons of clinicopathological features between RSCC and LSCC only included stage I‐III diseases and they concluded that RSCC had more advanced stages 5, 15, 20, 21, 22. Here, we pointed out that actually RSCC patients had less metastasis diseases than LSCC. The complicated relationship between clinicopathologic features and tumor location in colon cancer might partly explain the controversial results of survival comparison between RSCC and LSCC patients 4, 5, 12, 13, 14, 15, 16. Some found that RSCC had better survival than LSCC 11, 18, 19, 20, 23. Other studies considered that location of colon had no relationship with survival 15, 24. Thinking about the controversial results in the literatures, we hypothesized that the comparison of survival between RSCC and LSCC might vary in different situations.

Here, we firstly reported that RSCC had better prognosis than LSCC in mucinous adenocarcinoma/signet ring cell carcinoma patients. Except for this, RSCC also had better prognosis than LSCC in stage II diseases when the number of resected lymph nodes was over 11, consistent with previous reports 15, 19, 21. To further understand the above findings, we compared the clinicopathologic features between RSCC and LSCC in stage II patients and mucinous adenocarcinoma/signet ring cell carcinoma patients. We found that the clinicopathologic features were similar in the subgroups and in the whole population. It seemed that the better survival of RSCC patients in the above two situations was more likely related to tumor biology. Previous study showed that survival in stage II/III colorectal cancer was independently predicted by microsatellite instability (MSI), but not by specific driver mutations 25. MSI is predominantly seen in RSCC (about 25%) 3, while <5% in LSCC 6. Mucinous adenocarcinoma was more common in RSCC and was reported to have more MSI than nonmucinous adenocarcinoma 26. High MSI is related to a better overall survival 27, 28, 29 despite the fact that the effect of adjuvant chemotherapy, especially 5‐fluorouracil, is reduced in patients with MSI 30. We hypothesized that MSI was the major contributor to the reverse mortality between RSCC and LSCC in stage II and mucinous adenocarcinoma/signet ring cell carcinoma patients. Further researches are needed to confirm our hypothesis.

In the multivariate analysis, we found that location was an independent prognostic factor in the whole population. LSCC had lower mortality rate than RSCC with a hazard ratio of 0.87. There were other studies using the SEER database or SEER‐Medicare database trying to explore the role of location on survival. In Weiss JM's study, they found no difference in 5‐year mortality between RSCC and LSCC patients 15. Their study was limited in the patients’ age and stage. All the patients were 66 years and older and they only enrolled stage I to III patients. LSCC patients were younger and had more stage IV diseases 13. In our studies, we showed that LSCC had better prognosis than RSCC in younger patients and also stage IV patients. After excluding patients whose prognosis favoring LSCC, it was not hard to understand why no survival difference was found in Weiss JM's study. Meguid et al. 19 analyzed patients who underwent surgical resection for invasive colon adenocarcinoma using the SEER database between 1988 and 2003, and found that RSCC had worse prognosis than LSCC patients, which was similar to our result. This study was also limited to patients’ selection. Only patients who received surgical resection were considered. The patients’ selection in our present study was more close to real world.

Potential limitations of our study should be taken into consideration. Unmeasured factors in SEER database, such as chemotherapy and tumor biology, including MSI status might play roles in patient outcome. Recent reports showed that RSCC and LSCC even had different response to the anti‐Epidermal growth factor receptor (EGFR) and anti‐Vascular endothelial growth factor (VEGF) monoclonal antibody 31. We could not fully evaluate the impact of chemotherapy and target therapy on survival of RSCC and LSCC patients.

In conclusion, the relationship between survival and tumor location in colon cancer was not straightforward. There is a need for further subdivisions when analyzing the survival difference between RSCC and LSCC. We found that RSCC patients had better prognosis than LSCC in stage II disease or mucinous adenocarcinoma/signet ring cell carcinoma patients.

## Conflicts of Interests

None.

## Supporting information


**Appendix S1.** Clinicopathologic features of stage II patients between RSCC and LSCC patients
**Appendix S2.** Clinicopathologic features of mucinous adenocarcinoma and signet ring cell carcinoma between RSCC and LSCC patients
