# Supplementary material for: Comparison of survival between right‐sided and left‐sided colon cancer in different situations
Source: Cancer Med. 2018 Mar 13;7(4):1141–50. doi: 10.1002/cam4.1401 (PMC5911618; doi:10.1002/cam4.1401)
Supplement: Supplementary file 1 — Appendix S1. Clinicopathologic features of stage II patients between RSCC and LSCC patients Appendix S2. Clinicopathologic features of mucinous adenocarcinoma and signet ring cell carcinoma between RSCC and LSCC patients [file CAM4-7-1141-s001.docx]

Comparison of survival between right-sided and left-sided colon cancer in different situations

Appendix Tables – Online Only

Appendix 1. Clinicopathologic features of stage II patients between RSCC and LSCC patients

|  | Right-sided colon (%) | Left-sided colon (%) | *P* values |
| --- | --- | --- | --- |
| Gender |  |  |  |
| Male | 13,741 (45.52) | 9,000 (52.76) |  |
| Female | 16,445 (54.48) | 8,058 (47.24) | <0.001 |
| Age (Mean±SD) | 70.97±13.30 | 66.60±13.56 | <0.001 |
| Ethnicity |  |  |  |
| Caucasian | 24,772 (82.06) | 13,267 (77.78) |  |
| African American | 3,363 (11.14) | 1,977 (11.59) |  |
| Asian | 1,674 (5.55) | 1,526 (8.94) |  |
| Others | 377 (1.25) | 288 (1.69) | <0.001 |
| Married status |  |  |  |
| Married | 15,428 (51.11) | 9,143 (53.60) |  |
| Unmarried | 13,465 (44.61) | 7,199 (42.20) |  |
| Unknown | 1,293 (4.28) | 716 (4.20) | <0.001 |
| AJCC 6^th^ T stage |  |  |  |
| T3 | 26,292 (87.10) | 14,360 (84.18) |  |
| T4 | 3,894 (12.90) | 2,698 (15.82) | <0.001 |
| Histology |  |  |  |
| Other adenocarcinoma | 25,501 (84.48) | 15,674 (91.89) |  |
| Mucinous adenocarcinoma | 4,427 (14.67) | 1,336 (7.83) |  |
| Signet ring cell | 258 (0.85) | 48 (0.28) | <0.001 |
| Grade |  |  |  |
| Well differentiated | 2,175 (7.21) | 1,335 (7.83) |  |
| Moderately differentiated | 21,292 (70.54) | 13,468 (78.95) |  |
| Poorly differentiated | 5,470 (18.12) | 1,756 (10.29) |  |
| Undifferentiated | 605 (2.00) | 174 (1.02) |  |
| Unknown | 644 (2.13) | 325 (1.91) | <0.001 |
| Lymph node resected (Mean±SD) | 19.22±11.37 (17) | 16.71±11.67 (15) | <0.001 |
| Tumor size (Mean±SD, mm) | 55.45±38.03 (50) | 50.73±31.81 (45) | <0.001 |
| Surgery |  |  |  |
| Yes | 30,008 (99.41) | 16,895 (99.04) |  |
| No | 178(0.59) | 163 (0.96) | <0.001 |
| Radiation |  |  |  |
| Yes | 315 (1.04) | 637 (3.73) |  |
| No | 29,689 (98.35) | 16,279 (95.44) |  |
| Unknown | 182 (0.61) | 142 (0.83) | <0.001 |

Abbreviation: RSCC: Right-sided colon cancer; LSCC: Left-sided colon cancer; SD: standard deviation; AJCC: American Joint Committee on Cancer.

Appendix 2. Clinicopathologic features of mucinous adenocarcinoma and signet ring cell carcinoma between RSCC and LSCC patients

|  | Right-sided colon (%) | Left-sided colon (%) | *P* values |
| --- | --- | --- | --- |
| Gender |  |  |  |
| Male | 5,789 (44.53) | 2,459 (54.63) |  |
| Female | 7,210 (55.47) | 2,042 (45.37) | <0.001 |
| Age (Mean±SD) | 68.93±14.29 | 63.92±14.99 | <0.001 |
| Ethnicity |  |  |  |
| Caucasian | 10,747 (82.68) | 3,561 (79.12) |  |
| African American | 1,469 (11.30) | 566 (12.57) |  |
| Asian | 652 (5.02) | 316 (7.02) |  |
| Others | 131 (1.00) | 58 (1.29) | <0.001 |
| Married status |  |  |  |
| Married | 6,720 (51.70) | 2,441 (54.23) |  |
| Unmarried | 5,819 (44.76) | 1,888 (41.95) |  |
| Unknown | 460 (3.54) | 172 (3.82) | 0.004 |
| AJCC 6^th^ TNM stage |  |  |  |
| I | 1,559 (11.99) | 493 (10.95) |  |
| II | 4,685 (36.04) | 1,384 (30.75) |  |
| III | 4,291 (33.01) | 1,450 (32.22) |  |
| IV | 2,464 (18.96) | 1,174 (26.08) | <0.001 |
| AJCC 6^th^ T stage |  |  |  |
| T0 | 2 (0.02) | 1 (0.02) |  |
| T1 | 534 (4.10) | 308 (6.84) |  |
| T2 | 1,506 (11.59) | 351 (7.81) |  |
| T3 | 7,676 (59.05) | 2,392 (53.14) |  |
| T4 | 3,024 (23.26) | 1,291 (28.68) |  |
| TX | 257 (1.98) | 158 (3.51) | <0.001 |
| AJCC 6^th^ N stage |  |  |  |
| N0 | 6,679 (51.38) | 2,143 (47.61) |  |
| N1 | 3,223 (24.79) | 1,169 (25.97) |  |
| N2 | 2,906 (22.36) | 1,070 (23.77) |  |
| NX | 191 (1.47) | 119 (2.65) | <0.001 |
| AJCC 6^th^ M stage |  |  |  |
| M0 | 10,535 (81.04) | 3,327 (73.92) |  |
| M1 | 2,464 (18.96) | 1,174 (26.08) | <0.001 |
| Grade |  |  |  |
| Well differentiated | 1,028 (7.91) | 538 (11.95) |  |
| Moderately differentiated | 7,270 (55.93) | 2,364 (52.52) |  |
| Poorly differentiated | 3,279 (25.23) | 971 (21.57) |  |
| Undifferentiated | 431 (3.32) | 141 (3.13) |  |
| Unknown | 991 (7.61) | 487 (10.83) | <0.001 |
| Lymph node resected (Mean±SD) | 19.13±13.99 | 16.12±14.95 | <0.001 |
| Lymph node positive (Mean±SD) | 8.01±22.54 | 12.71±29.21 | <0.001 |
| Tumor size (Mean±SD, mm) | 59.76±40.30 | 56.8±34.85 | <0.001 |
| Surgery |  |  |  |
| Yes | 12,499 (96.15) | 4,199 (93.29) |  |
| No | 496 (3.82) | 300 (6.67) |  |
| Unknown | 4 (0.03) | 2 (0.04) | <0.001 |
| Radiation |  |  |  |
| Yes | 236 (1.82) | 185 (4.11) |  |
| No | 12,659 (97.38) | 4,270 (94.87) |  |
| Unknown | 104 (0.80) | 46 (1.02) | <0.001 |

Abbreviation: RSCC: Right-sided colon cancer; LSCC: Left-sided colon cancer; SD: standard deviation; AJCC: American Joint Committee on Cancer; TNM: Tumor-Node-Metastasis.
